# Machine learning for the prediction of all-cause mortality in patients with sepsis-associated acute kidney injury during hospitalization

**DOI:** 10.3389/fimmu.2023.1140755

**Published:** 2023-04-03

**Authors:** Hongshan Zhou, Leping Liu, Qinyu Zhao, Xin Jin, Zhangzhe Peng, Wei Wang, Ling Huang, Yanyun Xie, Hui Xu, Lijian Tao, Xiangcheng Xiao, Wannian Nie, Fang Liu, Li Li, Qiongjing Yuan

**Affiliations:** ^1^ Department of Nephrology, Xiangya Hospital of Central South University, Changsha, Hunan, China; ^2^ Department of Pediatrics, The Third Xiangya Hospital, Central South University, Changsha, China; ^3^ College of Engineering and Computer Science, Australian National University, Canberra, ACT, Australia; ^4^ Critical Care Medicine, The Third Xiangya Hospital, Central South University, Changsha, Hunan, China; ^5^ Organ Fibrosis Key Lab of Hunan Province, Central South University, Changsha, Hunan, China; ^6^ National International Joint Research Center for Medical Metabolomices, Xiangya Hospital, Central South University, Changsha, Hunan, China; ^7^ Health Management Center, Xiangya Hospital of Central South University, Changsha, Hunan, China; ^8^ Critical Care Medicine, Xiangya Hospital of Central South University, Changsha, Hunan, China; ^9^ National Clinical Medical Research Center for Geriatric Diseases, Xiangya Hospital of Central South University, Changsha, Hunan, China

**Keywords:** sepsis, acute kidney injury, mortality, predictive model, machine learning

## Abstract

**Background:**

Sepsis-associated acute kidney injury (S-AKI) is considered to be associated with high morbidity and mortality, a commonly accepted model to predict mortality is urged consequently. This study used a machine learning model to identify vital variables associated with mortality in S-AKI patients in the hospital and predict the risk of death in the hospital. We hope that this model can help identify high-risk patients early and reasonably allocate medical resources in the intensive care unit (ICU).

**Methods:**

A total of 16,154 S-AKI patients from the Medical Information Mart for Intensive Care IV database were examined as the training set (80%) and the validation set (20%). Variables (129 in total) were collected, including basic patient information, diagnosis, clinical data, and medication records. We developed and validated machine learning models using 11 different algorithms and selected the one that performed the best. Afterward, recursive feature elimination was used to select key variables. Different indicators were used to compare the prediction performance of each model. The SHapley Additive exPlanations package was applied to interpret the best machine learning model in a web tool for clinicians to use. Finally, we collected clinical data of S-AKI patients from two hospitals for external validation.

**Results:**

In this study, 15 critical variables were finally selected, namely, urine output, maximum blood urea nitrogen, rate of injection of norepinephrine, maximum anion gap, maximum creatinine, maximum red blood cell volume distribution width, minimum international normalized ratio, maximum heart rate, maximum temperature, maximum respiratory rate, minimum fraction of inspired O_2_, minimum creatinine, minimum Glasgow Coma Scale, and diagnosis of diabetes and stroke. The categorical boosting algorithm model presented significantly better predictive performance [receiver operating characteristic (ROC): 0.83] than other models [accuracy (ACC): 75%, Youden index: 50%, sensitivity: 75%, specificity: 75%, F1 score: 0.56, positive predictive value (PPV): 44%, and negative predictive value (NPV): 92%]. External validation data from two hospitals in China were also well validated (ROC: 0.75).

**Conclusions:**

After selecting 15 crucial variables, a machine learning-based model for predicting the mortality of S-AKI patients was successfully established and the CatBoost model demonstrated best predictive performance.

## Introduction

Sepsis, which is one of the principal causes of mortality worldwide and affects more than 19 million people every year (1–3), is defined as a sequential fatal organ dysfunction after infection with a dysregulated host response by the Third International Consensus Definitions for Sepsis and Septic Shock (Sepsis-3). Similarly, the Kidney Disease: Improving Global Outcomes (KDIGO) group integrated previous diagnostic criteria and proposed an international consensus for acute kidney injury (AKI) to be defined as (i) an increase in SCr level by more than 26.5 μmol/L (0.3 mg/dl) within 48 h; (ii) an increase in SCr level by more than 1.5 times the baseline (confirmed or presumed to occur within 7 days); and (iii) urine volume <0.5 ml/(kg·h) lasting for more than 6 h (4). In critically ill patients, the main cause of AKI has been considered to be sepsis for a long time, and 45%–70% of AKI patients are considered to have sepsis (5). Thus, sepsis-associated acute kidney injury (S-AKI) should be defined as a syndrome that meets the Sepsis-3 and KDIGO criteria simultaneously (6).

The epidemiology of S-AKI has not been fully clarified probably because of uncoordinated epidemiology of sepsis and AKI criteria, but the global incidence is estimated to be 6 million cases annually (6). The mortality of S-AKI was reported to be 45.99% in the intensive care unit (ICU) (7), and a retrospective cohort study discovered that S-AKI was correlated with a significantly higher mortality rate compared to sepsis without AKI (71.7% vs. 21.3%) (8). At present, many studies have shown that S-AKI imposed a heavy burden on patients. In a review, Hoste et al. summarized that the occurrence of AKI was related to the severity of sepsis and that S-AKI was responsible for the increase in disease acuity and burden of organ dysfunction (9). Bagshaw et al. conducted an observational cohort study spanning multiple nations and centers, which reported that S-AKI was associated with a high-crude in-hospital case fatality rate (51.8%) (5). Furthermore, a multicenter retrospective cohort study in China concluded that sepsis resulted in 32.0% of hospital-acquired AKI and 15.2% of community-acquired AKI. In addition, AKI was correlated with high mortality, longer length of stay, and heavier daily expenses while in the hospital (10). Additionally, an observational study of 618 ICU patients with AKI, the Program to Improve Care in Acute Renal Disease (PICARD), revealed that the in-hospital mortality rate of S-AKI was noticeably high, regardless of sepsis occurring before AKI (48%) or after AKI (44%) (11).

Considering that S-AKI patients experience high morbidity and mortality, the precise prediction of their prognosis is necessary. Novel biomarkers like tissue inhibitor of metalloproteinases-2 (TIMP-2), neutrophil gelatinase-associated lipocalin (NGAL), and insulin-like growth factor binding protein-7 (IGFBP-7) have been evaluated to forecast the prognosis of S-AKI; however, their sensitivity has not been verified in large multicenter studies (12). Conventional scoring systems of severity, such as Sequential Organ Failure Assessment (SOFA) and Acute Physiology and Chronic Health Evaluation II (APACHE II), have been widely used in the ICU to predict outcomes. Regrettably, they lack discrimination and prediction accuracy, and external validation is required before application to S-AKI cohorts (13). Consequently, it is essential to establish a new model that efficiently and accurately predicts the outcomes of S-AKI.

As a novel technology, machine learning has been utilized in various medical fields owing to its ability to develop robust risk models and improve prediction power (14, 15). The accuracy of predicting the occurrence of S-AKI utilizing machine learning has been confirmed (16–18). However, this radical new technology has not been applied to predict the mortality of patients with S-AKI, which is equally noteworthy. Gradient boosted decision trees (GBDTs) are powerful machine learning ensemble techniques, particularly when massive amounts of data are involved in classification and regression tasks. As one of the GBDT families, CatBoost is perfectly suited to processing categorical, heterogeneous data (19). Since its debut, CatBoost has been used in some medical studies and demonstrated its excellent predictive ability.

This study aimed to identify the risk factors associated with mortality in patients with S-AKI and develop a machine learning model to predict death in hospitals on the basis of primary research emphasizing the prediction of occurrence. The performance of this machine learning model was compared with 10 other machine learning models to validate the superiority of the proposed model.

## Materials and methods

### Study subjects

**Figure 1:**
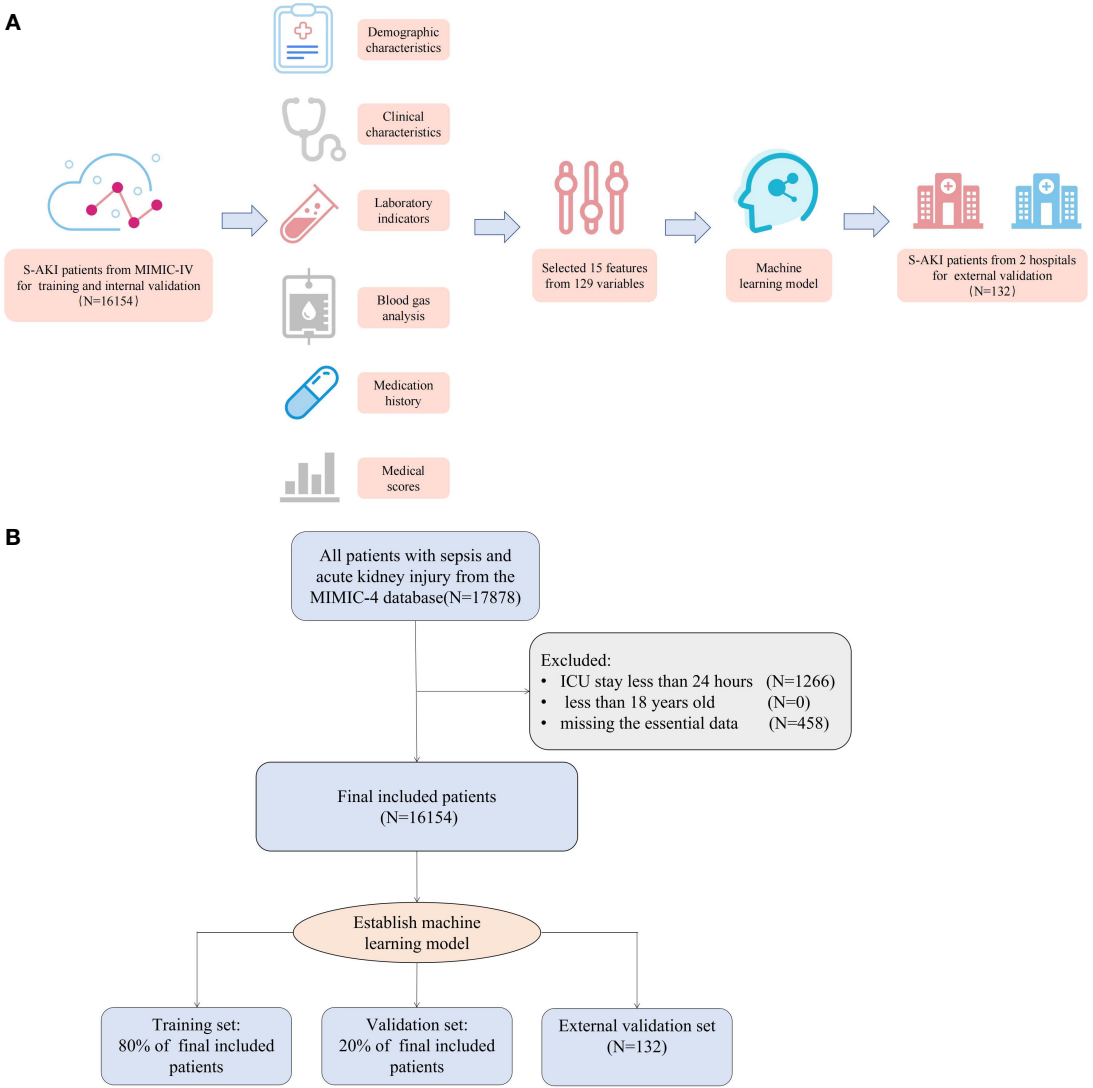
**(A)** The workflow of the study. **(B)** The algorithm chart of the study.

The Medical Information Mart for Intensive Care IV (MIMIC-IV) is a database containing patient data from all ICU and emergency departments at Beth Israel Deaconess Medical Center from 2008 to 2019. The contents of the database include basic patient information, diagnosis, clinical data, and medication records, among others. We extracted the data of patients with sepsis and AKI after admission from the MIMIC-IV database as training and validation sets. Then, we collected the data of patients with sepsis and AKI in the ICU of Xiangya Hospital (from 2015 to 2022) and Third Xiangya Hospital (from 2022) of Central South University, Changsha, China as an external validation set (**Figure 1**).

According to the KDIGO guidelines, AKI is characterized by one or more of the following: (i) an increase in SCr level by more than 26.5 μmol/L (0.3 mg/dl) within 48 h; (ii) an increase in SCr level by more than 1.5 times the baseline (confirmed or presumed to occur within 7 days); and (iii) urine volume <0.5 ml/(kg·h) lasting for more than 6 h. According to the Third International Consensus Definitions for Sepsis and Septic Shock (Sepsis-3), sepsis is characterized by life-threatening organ dysfunction as a result of infection coupled with an impaired host response. According to the SOFA, organ dysfunction is a change in the total SOFA score of 2 points caused by infection. As part of this study, patients who were younger than 18 years of age, had stayed in the ICU for less than 24 h, and missed essential data were excluded. We used multiple imputations to supplement the missing values of patients. The death group is composed of patients who died in the hospital, and the alive group consists of patients who did not die during hospitalization.

According to the ethical standards of the responsible committee on human experimentation in China and to the Helsinki Declaration of 1975, all procedures in this study were conducted in accordance with the ethical standards of the responsible committee. The study was initiated under the guidance of Transparent Reporting of a Multivariable Prediction Model for Individual Prognosis or Diagnosis (TRIPOD) (**Supplementary Figure 1**). The Xiangya Hospital of Central South University Ethics Committee reviewed and approved this study on 27 April 2022 (protocol number 202204101), which used machine learning to predict all-cause mortality among patients with S-AKI while hospitalized.

### Study design and data collection

We collected 129 variables within 24 h of admission. The collected variables included patients’ basic information, diagnosis, medication records, clinical data such as temperature, blood pressure, concomitant disease, laboratory indicators, urine output (24-h urine volume after diagnosis of S-AKI), injection rate of norepinephrine (initial concentration of norepinephrine after diagnosis of S-AKI), and commonly used scores such as Simplified Acute Physiology Score II (SAPS-II), SOFA score, and Glasgow Coma Scale (GCS). The external validation set was derived from the electronic health record systems of Xiangya Hospital and Third Xiangya Hospital. The data were collected by two authors (LL and HZ). Data collected by different hospitals were converted and unified. As an example, the injection rate of norepinephrine at 1 mcg/kg/min equaled 1 μg/kg/min. The concentration of creatinine in the blood was 88.4 μmol/L per mg/dl.

### Statistical analysis

As appropriate, continuous variables were compared between the death and alive groups using either Student’s *t*-test or the rank-sum test. A chi-square test or Fisher’s exact test was used to compare categorical variables.

Then, the data were standardized such that the mean value was 0 and the standard deviation was 1. The K-nearest neighbor (KNN) algorithm was used to impute missing values. Next, the dataset was randomly split into a training set (80%) and a validation set (20%). On the training set, the recursive feature elimination (RFE) algorithm was utilized to identify crucial variables, and we developed a machine learning model based on categorical boosting (CatBoost) (20). Basically, RFE is a way of selecting features that recursively fit a model derived from smaller feature sets until a given termination criterion is reached. A feature’s importance in the trained model is graded in each loop. In an RFE model, dependencies and collinearities are eliminated by recursively eliminating the lowest-priority feature. As a final step, the most important features were screened out, and the CatBoost model was developed based on the final set of features. Other features were not included because they only brought a small increment in the area under the receiver operating characteristic (AUROC) curve but significantly increased the difficulty of model applications. The trained model was validated on the validation set, and the AUROC curve was calculated correspondingly.

This study compared 10 other machine learning models to the proposed one, namely, KNN, AdaBoost, multilayer perceptron (MLP), support vector machine (SVM), logistic regression (LR), NaiveBayes, gradient boosting decision tree (GBDT), random forest, light gradient boosting (LightGBM), and extreme gradient boosting (XGBoost). These models were also developed on the training set and validated on the validation set. AUROC curves were compared between these models and our CatBoost model. Additionally, other performance measures were examined, such as accuracy (ACC), Youden index, sensitivity, specificity, F1 score, positive predictive value (PPV), and negative predictive value (NPV).

To explain the model, the SHapley Additive exPlanations (SHAP) package in Python was used. A game-theoretic approach is used by the SHAP package to interpret the output of the machine learning model (21). The model was able to connect optimal credit allocation to local explanations for each prediction sample. Two cases were analyzed by using SHAP values to examine model interpretability. The statistical analyses that were carried out in the present study were performed using Python (version 3.7.6); a significance level of *p* < 0.05 was considered to be statistically significant.

## Results

### Study population

There were 16,154 patients included in the MIMIC-IV set, and relevant information of the cohort can be viewed in **Table 1**. The average age of the patients was 67.7 years, men accounted for 42.3%, and the average body mass index (BMI) was 30.9. In the cohort, 20.5% of the patients died in the hospital, and their length of stay in the ICU was 3.7 days, longer than that of patients in the alive group. Information of external validation cohort is shown in **Supplementary Table 1** and overall workflow and algorithm chart are shown in **Figure 1**.

**Table 1:** Most of the variables that differ between the two groups in the MIMIC-IV set.

Variable		All (*n* = 16,154)	Alive group (*n* = 12,836)	Death group (*n* = 3,318)	*p*-Value
*N*		16,154	12,836	3,318	
Charlson Index, median [Q1,Q3]		6.0 [4.0,8.0]	6.0 [4.0,8.0]	7.0 [5.0,9.0]	<0.001
Age, mean (SD)		67.7 (15.2)	67.1 (15.2)	70.3 (14.8)	<0.001
Gender, *n* (%)	F	6,836 (42.3)	5,362 (41.8)	1,474 (44.4)	0.006
	M	9,318 (57.7)	7,474 (58.2)	1,844 (55.6)	
Ethnicity, *n* (%)	Asian	377 (2.3)	294 (2.3)	83 (2.5)	<0.001
	Black	1,733 (10.7)	1,421 (11.1)	312 (9.4)	
	Hispanic	538 (3.3)	443 (3.5)	95 (2.9)	
	Other	2,586 (16.0)	1,839 (14.3)	747 (22.5)	
	White	10,920 (67.6)	8,839 (68.9)	2,081 (62.7)	
Liver disease, *n* (%)		3,253 (20.1)	2,096 (16.3)	1,157 (34.9)	<0.001
Stroke, *n* (%)		1,014 (6.3)	666 (5.2)	348 (10.5)	<0.001
BMI, mean (SD)		30.9 (8.8)	31.2 (8.7)	29.5 (8.7)	<0.001
SAPS-II, median [Q1,Q3]		42.0 [34.0,52.0]	40.0 [32.0,49.0]	54.0 [44.0,66.0]	<0.001
SOFA, median [Q1,Q3]		6.0 [4.0,9.0]	5.0 [4.0,8.0]	9.0 [6.0,12.0]	<0.001
GCS, median [Q1,Q3]		15.0 [13.0,15.0]	15.0 [13.0,15.0]	15.0 [12.0,15.0]	<0.001
Heart rate max, mean (SD)		106.1 (21.6)	104.3 (20.5)	113.0 (23.9)	<0.001
Heart rate min, mean (SD)		72.0 (15.9)	71.3 (15.0)	74.6 (18.9)	<0.001
Respiratory rate max, mean (SD)		28.7 (6.7)	28.1 (6.4)	30.7 (7.1)	<0.001
Respiratory rate min, mean (SD)		12.5 (3.9)	12.2 (3.7)	13.4 (4.5)	<0.001
MBP max, mean (SD)		105.0 (28.7)	104.7 (27.3)	106.3 (33.6)	0.016
MBP min, mean (SD)		54.6 (13.4)	55.9 (12.5)	49.7 (15.3)	<0.001
SBP max, mean (SD)		146.4 (23.9)	147.2 (23.3)	143.4 (25.7)	<0.001
SBP min, mean (SD)		85.9 (16.4)	87.6 (15.4)	79.2 (18.3)	<0.001
PaO_2_ max, median [Q1,Q3]		174.0 [104.0,321.0]	188.0 [109.0,343.0]	144.0 [94.0,227.0]	<0.001
PaO_2_ min, median [Q1,Q3]		84.0 [65.0,111.0]	87.0 [68.0,115.0]	73.0 [56.0,96.0]	<0.001
SpO_2_ max, median [Q1,Q3]		100.0 [100.0,100.0]	100.0 [100.0,100.0]	100.0 [100.0,100.0]	<0.001
SpO_2_ min, median [Q1,Q3]		92.0 [90.0,95.0]	93.0 [90.0,95.0]	91.0 [86.0,94.0]	<0.001
Temperature max, mean (SD)		37.5 (0.8)	37.5 (0.8)	37.4 (1.0)	<0.001
Temperature min, mean (SD)		36.2 (0.8)	36.3 (0.7)	36.0 (1.1)	<0.001
AST max, median [Q1,Q3]		54.0 [28.0,161.0]	47.0 [26.0,121.0]	91.0 [37.0,345.0]	<0.001
AST min, median [Q1,Q3]		48.0 [26.0,123.0]	42.0 [24.0,97.0]	72.0 [32.0,217.0]	<0.001
PTT max, median [Q1,Q3]		34.4 [29.3,46.4]	33.4 [28.8,42.7]	40.9 [31.7,64.5]	<0.001
PTT min, median [Q1,Q3]		30.7 [27.1,36.7]	30.0 [26.8,35.1]	34.4 [28.8,43.6]	<0.001
Platelet max, median [Q1,Q3]		189.0 [135.0,257.0]	190.0 [139.0,255.0]	183.0 [114.0,268.0]	<0.001
Platelet min, median [Q1,Q3]		160.0 [107.0,226.0]	162.0 [112.0,226.0]	151.0 [84.0,228.0]	<0.001
RBC max, mean (SD)		3.6 (0.7)	3.6 (0.7)	3.5 (0.8)	<0.001
RBC min, mean (SD)		3.2 (0.7)	3.3 (0.7)	3.2 (0.8)	<0.001
WBC max, median [Q1,Q3]		13.5 [9.5,18.6]	13.2 [9.4,18.0]	15.0 [10.0,21.2]	<0.001
WBC min, median [Q1,Q3]		10.4 [7.3,14.5]	10.2 [7.2,13.8]	11.7 [7.4,17.0]	<0.001
RDW max, mean (SD)		15.9 (2.5)	15.6 (2.4)	16.9 (2.8)	<0.001
RDW min, mean (SD)		15.5 (2.4)	15.3 (2.3)	16.5 (2.7)	<0.001
Glucose max, median [Q1,Q3]		143.0 [115.0,194.0]	140.0 [114.0,186.0]	162.0 [122.0,225.2]	<0.001
Glucose min, median [Q1,Q3]		115.0 [95.0,141.0]	115.0 [96.0,139.0]	114.0 [88.0,148.0]	0.017
Lactate max, median [Q1,Q3]		2.3 [1.5,3.8]	2.2 [1.5,3.3]	3.5 [1.9,7.2]	<0.001
Lactate min, median [Q1,Q3]		1.6 [1.2,2.3]	1.5 [1.1,2.1]	2.2 [1.4,3.8]	<0.001
BUN max, median [Q1,Q3]		27.0 [18.0,45.0]	25.0 [17.0,41.0]	38.0 [25.0,58.0]	<0.001
BUN min, median [Q1,Q3]		23.0 [15.0,39.0]	22.0 [15.0,35.0]	32.0 [21.0,52.0]	<0.001
Creatinine max, median [Q1,Q3]		1.4 [0.9,2.5]	1.3 [0.9,2.2]	1.9 [1.2,3.1]	<0.001
Creatinine min, median [Q1,Q3]		1.2 [0.8,2.1]	1.1 [0.8,1.9]	1.6 [1.0,2.6]	<0.001
Urine output, median [Q1,Q3]		1,040.0 [537.0,1,665.0]	1,150.0 [675.0,1,760.0]	605.0 [186.0,1,110.0]	<0.001
RRT, *n* (%)		1,633 (10.1)	1,135 (8.8)	498 (15.0)	<0.001
IMV, *n* (%)		9,518 (58.9)	7,398 (57.6)	2,120 (63.9)	<0.001
Vasopressor support, *n* (%)		5,912 (36.6)	3,942 (30.7)	1,970 (59.4)	<0.001
Rate of norepinephrine, median [Q1,Q3]		0.0 [0.0,0.1]	0.0 [0.0,0.0]	0.1 [0.0,0.4]	<0.001
IMV durations, median [Q1,Q3]		0.4 [0.0,2.6]	0.2 [0.0,1.9]	1.6 [0.0,5.1]	<0.001
Hospital mortality, *n* (%)		3,318 (20.5)	0(0.0)	3,318 (100.0)	<0.001
Length of ICU stay, median [Q1,Q3]		3.0 [1.7,6.0]	2.9 [1.7,5.6]	3.7 [1.7,7.5]	<0.001
Length of hospital stay, median [Q1,Q3]		8.7 [5.2,15.1]	9.1 [5.8,15.7]	6.3 [2.4,13.3]	<0.001

SD, standard deviation; BMI, body mass index; SAPS-II, Simplified Acute Physiology Score II; SOFA, Sequential Organ Failure Assessment; GCS, Glasgow Coma Scale; MBP, mean blood pressure; SBP, systolic blood pressure; PaO_2_, partial pressure of oxygen; SpO_2_, saturation of pulse oxygen; AST, aspartate aminotransferase; PTT, partial thromboplastin time; RBC, red blood cell; WBC, white blood cell; RDW, red blood cell volume distribution width; BUN, blood urea nitrogen; RRT, renal replacement therapy; IMV, intermittent mandatory ventilation; ICU, intensive care unit.

### Key variables

**Figure 2:**
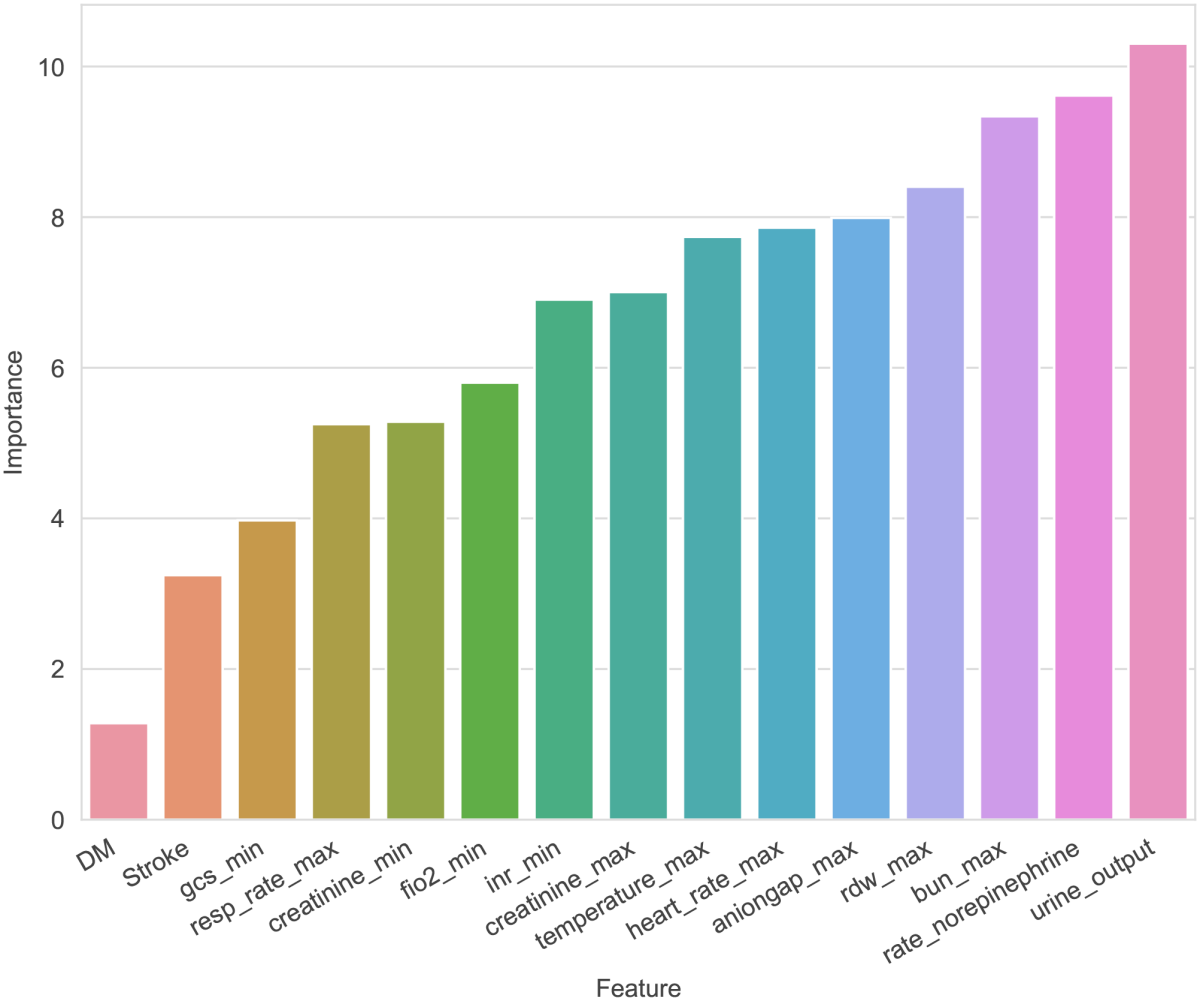
The importance of each feature to the machine learning model.

After utilizing the RFE algorithm, 15 essential variables were selected, namely, urine output, maximum blood urea nitrogen (BUN), rate of injection of norepinephrine, maximum anion gap, maximum creatinine, maximum red blood cell volume distribution width (RDW), minimum international normalized ratio (INR), maximum heart rate, maximum temperature, maximum respiratory rate, minimum fraction of inspired O_2_ (FiO_2_), minimum creatinine, minimum GCS score, and diagnosis of diabetes and stroke (**Figure 2**).

**Figure 3:**
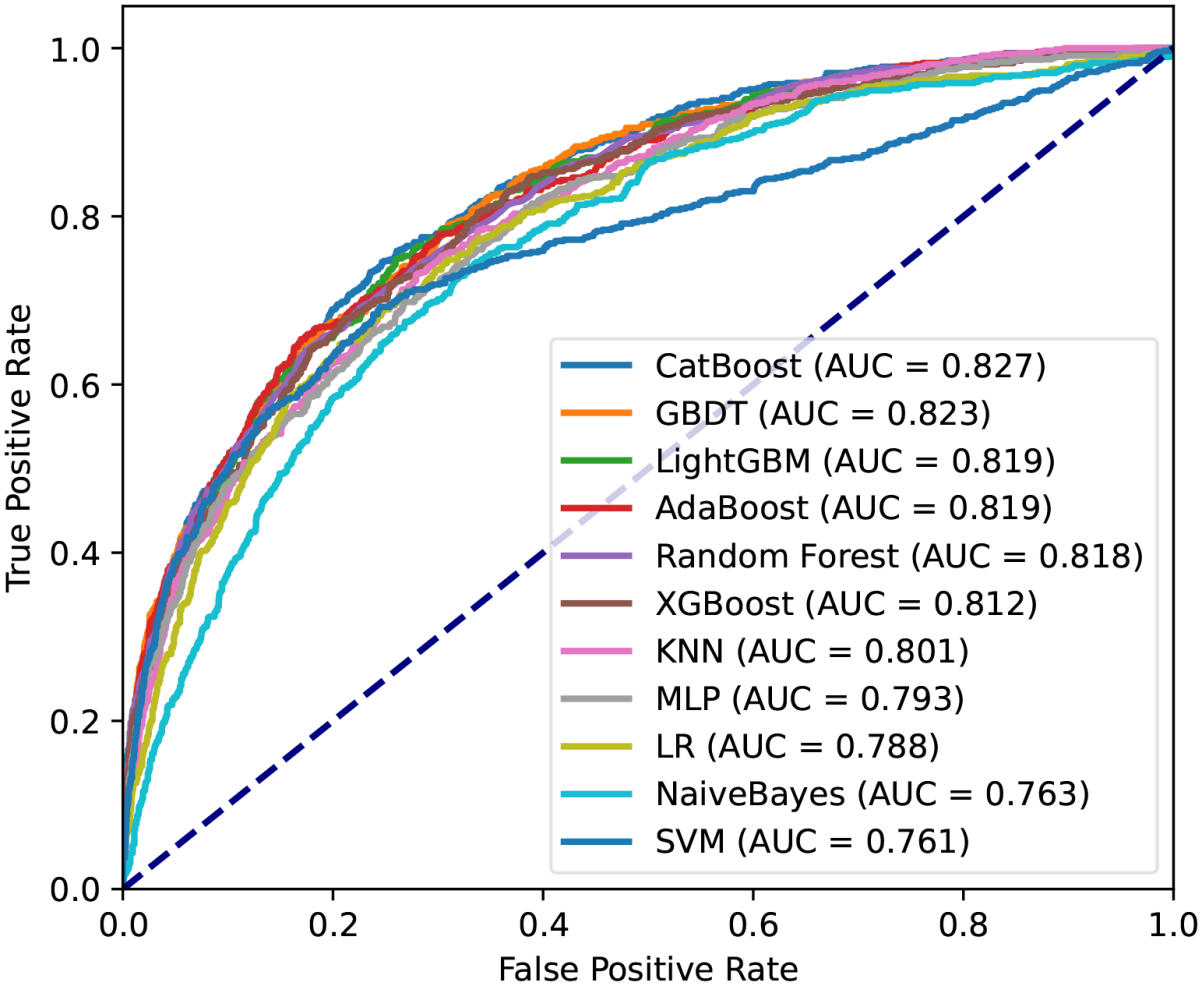
Receiver operating characteristic curves for the machine learning model and logistic regression in the training set. CatBoost, categorical boosting; GBDT, gradient boosting decision tree; LightGBM, light gradient boosting; AdaBoost, adaptive boosting; XGBoost, extremely gradient boosting; KNN, K-nearest neighbor; MLP, multilayer perceptron; LR, logistic regression. SVM, support vector machine.

Then, machine learning was used for predicting hospital death of patients. The AUC of the proposed CatBoost model was 0.827, which is shown in **Figure 3**. The CatBoost model markedly outperformed conventional LR (AUC: 0.788) and nine other machine learning models. As described in **Table 2**, the ACC, best cutoff, Youden index, sensitivity, specificity, F1 score, PPV, and NPV of the CatBoost model were 75%, 19.5%, 50%, 75%, 75%, 56%, 44%, and 92%, respectively. These indicators of LR were 73%, 20.1%, 44%, 71%, 74%, 52%, 41%, and 90%, respectively. In addition, the ROC curve of the validation set reached 0.75, indicating the good applicability of our model (**Supplementary Figure 2**). To compare with the conventional scoring system, a CatBoost model for the SOFA score was made, and the results show that the prediction ability of SOFA is inferior to the proposed model in the training and validation set (**Supplementary Figure 3**). As AST was almost double in the death group, and in the raw data, the number of patients with AST greater than 45 U/L was almost equal to the number of patients with liver disease. Therefore, a CatBoost model was also established to conduct a liver disease subgroup analysis that also demonstrates a good prediction power on the mortality of S-AKI among these subgroup patients (**Supplementary Figure 4**).

**Table 2:** Performance of machine learning models.

Model	AUC	ACC (%)	Best cutoff	Youden index (%)	Sensitivity (%)	Specificity (%)	F1 score	PPV (%)	NPV (%)
CatBoost	0.83	75	0.195	50	75	75	0.56	44	92
GBDT	0.82	71	0.16	48	79	69	0.53	40	93
LightGBM	0.82	74	0.183	49	75	74	0.55	43	92
AdaBoost	0.82	79	0.494	48	65	83	0.57	51	90
Random Forest	0.82	78	0.28	47	66	81	0.55	48	90
XGBoost	0.81	77	0.204	47	68	79	0.55	46	90
KNN	0.8	72	0.176	45	73	72	0.52	41	91
MLP	0.79	73	0.162	43	70	73	0.52	41	90
LR	0.79	73	0.201	44	71	74	0.52	41	90
NaiveBayes	0.76	68	0.092	41	74	67	0.49	37	91
SVM	0.76	74	0.149	45	69	75	0.53	43	90

CatBoost, categorical boosting; GBDT, gradient boosting decision tree; LightGBM, light gradient boosting; AdaBoost, adaptive boosting; XGBooST, extremely gradient boosting; KNN, K-nearest neighbor; MLP, multilayer perceptron; LR, logistic regression. SVM, support vector machine; ACC, accuracy, PPV, positive predictive value; NPV, negative predictive value.

### Application of the model

**Figure 4:**
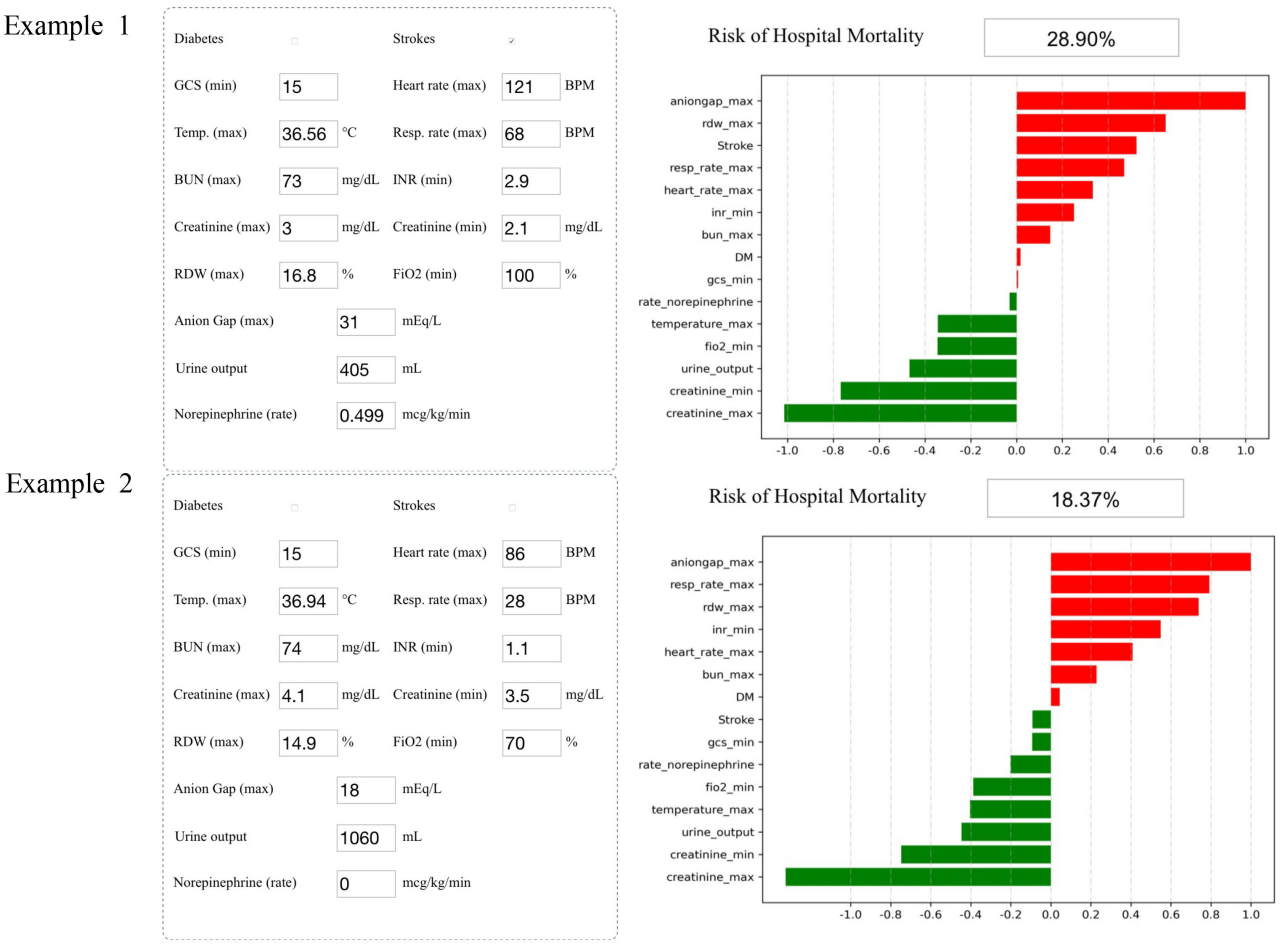
Two examples of website tool usage. Enter the values of 15 key variables to predict the risk of death and show the contribution of each value to the outcome. Example 1 has a higher risk of death, and example 2 may have a better prognosis.

Analyzing the integral cohort by the SHAP package showed the crucial variables for predicting death (**Figure 4**). Input the information of a patient into the model: history of stroke, minimum GCS score of 15, maximum heart rate of 121 beats per minute, maximum temperature of 36.56°C, maximum respiratory rate of 68 breaths per minute, maximum BUN level of 73 mg/dl, minimum INR of 2.9, maximum creatinine level of 3 mg/dl, minimum creatinine level of 2.1 mg/dl, maximum RDW of 16.8%, minimum FiO_2_ of 100%, maximum anion gap of 31 mEq/L, urine output of 405 ml/day, and a rate of injection of norepinephrine of 0.499 mcg/kg/min. The model showed that the risk of hospital mortality was 28.9% (higher than the best cutoff), suggesting that the patient had a high risk of death (Example 1, **Figure 4**). Input the information of another patient into the model: no history of stroke or diabetes, minimum GCS score of 15, maximum heart rate of 86 beats per minute, maximum temperature of 36.94°C, maximum respiratory rate of 28 breaths per minute, maximum BUN level of 74 mg/dl, minimum INR of 1.1, maximum creatinine level of 4.1 mg/dl, minimum creatinine level of 3.5 mg/dl, maximum RDW of 14.9%, minimum FiO_2_ of 70%, maximum anion gap of 18 mEq/L, urine output of 1,060 ml, and a rate of injection of norepinephrine of 0 mcg/kg/min. The probability of hospital mortality was predicted to be 18.37%, suggesting a good prognosis (Example 2, **Figure 4**).

## Discussion

Machine learning has been widely applied to solve medical and clinical problems, by which it has become a popular research topic. Based on their shortcomings, novel biomarkers and conventional scoring systems lack enough power to estimate the mortality of S-AKI patients (12, 13). In this article, we discussed whether machine learning improves the mortality prediction of S-AKI patients and then selected the model with the strongest prediction ability.

From the MIMIC-IV database used as a training set, 15 crucial variables were selected using the RFE algorithm. These variables are common in various clinical settings, which means information on them can be easily obtained, and the application of machine learning models will not be limited to a variable that is difficult to detect. Some studies have focused on the relative importance of each variable in predicting prognosis. For example, a retrospective study from a prospective cohort conducted by Sukmark et al. suggested that a lower GCS score was associated with in-ICU mortality with an adjusted odds ratio of 4.16 (3.10, 5.60) (22). Serum creatinine has been extensively utilized as a predictor in severity scores that assess renal function and adverse effects of renal dysfunction, such as SOFA and APACHE II. In addition, it has been reported that BUN is associated with multiorgan failure of ICU patients regardless of admission diagnosis, including kidney failure and long-term mortality (23). Sukmark also elaborated that BUN possibly reflected multiorgan failure better than serum creatinine (22). As mentioned before, some variables were found to be correlated with prognosis. However, few have put them into one prediction model and successfully quantified their ability to predict mortality.

After identifying these 15 variables, machine learning was applied to predict the mortality of patients during hospitalization. CatBoost is an open-source package and a new GBDT algorithm announced in 2017. Compared to other GBDT algorithms, it outperformed in handling categorical variables and reducing overfitting (24). To prove the efficiency of the CatBoost model, it was compared with 10 other machine learning models and SOFA. Satisfactorily, the proposed model significantly outperformed the others with an AUC of 0.827. Furthermore, we collected data from Xiangya Hospital and Third Xiangya Hospital, Central South University, China, to use as an external validation set. The ROC curve of the validation set was also as high as 0.754.

Compared with several other S-AKI-related clinical model studies (16–18), the innovation of this study is that the fourth edition of the MIMIC database used includes more patients from 2017 to 2019 than the third edition, with a larger amount of data and more recent data. In addition, in contrast to the related studies, emphasis was placed on predicting the mortality of S-AKI patients for the first time. Second, this study not only utilized data from the database but also collected data from other hospitals for validation, making the model more reliable. In addition, our training set is from Western countries, while the validation set is from China, indicating that the model has applicability among different populations. Moreover, instead of just using one machine learning algorithm to build the model, we compared multiple machine learning algorithms and selected the one that performed the best. Finally, since the chosen variables are easily accessible, the prediction model has a wide range of applications in areas with different medical levels.

However, our study has some limitations. First, the training set data originated from only one database, while the validation set data came from two hospitals in one region; thus, selection bias may have occurred. Even in view of this, the proposed model constructed by the MIMIC-IV database still passed the validation set from China, which, in turn, proved the superiority of our model. However, we must admit that more external validations are needed. Second, the variables were selected by the RFE algorithm, but the underlying mechanism was not discussed in our study.

As found in previous studies, S-AKI patients were treated with mechanical ventilation and vasoactive therapy with greater possibility (9), so was dialysis (70%) (11), which was simultaneously associated with a longer hospital stay (5). Prolonging hospital stays and expensive treatments mean an increasingly larger economic burden on patients and medical insurance. Meanwhile, it is sometimes challenging for clinicians to decide the priority treatment in the next step when condition deteriorates rapidly. Consequently, applying the CatBoost-based model to discern high-risk S-AKI patients and predict prognoses in a timely and accurate manner and providing clinicians with optimal treatment decision-making suggestions may help reduce these burdens. In conclusion, we hope that the proposed model will assist clinicians with better decision-making and allocating medical resources reasonably.

## Conclusions

This study demonstrates that predicting the mortality of S-AKI patients in the ICU is critical and that the CatBoost-based model we proposed outperformed conventional LR and nine other machine learning models. Further validations across diverse study centers will help verify the reliability and improve the validation efficiency of this model.

## Data availability statement

The original contributions presented in the study are included in the article/**Supplementary Material**. Further inquiries can be directed to the corresponding authors.

## Ethics statement

This study was reviewed and approved by the Ethics Committee of the Xiangya Hospital of Central South University on 27 April 2022 (protocol number 202204101).

## Author contributions

HZ: resources and writing—original draft; LPL: methodology, resources, validation, visualization, and writing—original draft; QZ: formal analysis, methodology, and validation; XJ: resources; ZP: investigation; WW: investigation; LH: investigation; YX: investigation; HX: supervision; LT: supervision; XX: supervision; WN: investigation; FL: review and editing; LL: review and editing; QY: review and editing and supervision. All authors contributed to the article and approved the submitted version.

## Supplementary material

Supplementary Figure 1 TRIPOD Checklist: Prediction Model Development.

Supplementary Figure 2 Receiver operating characteristic curves for the machine learning model and logistic regression in the validation set.

Supplementary Figure 3 Receiver operating characteristic curves for the machine learning model of SOFA score. **(A)** ROC of the training set. **(B)** ROC of the validation set.

Supplementary Figure 4 Receiver operating characteristic curves for the machine learning model of liver disease subgroup. **(A)** ROC of the training set. **(B)** ROC of the validation set.

Supplementary Table 1 Demographic and comorbidity information on the external validation cohort.
