# Supplementary figures and images for: Machine learning for the prediction of all-cause mortality in patients with sepsis-associated acute kidney injury during hospitalization
Source: Front Immunol. 2023 Apr 3;14:1140755. doi: 10.3389/fimmu.2023.1140755 (PMC10106833; doi:10.3389/fimmu.2023.1140755)

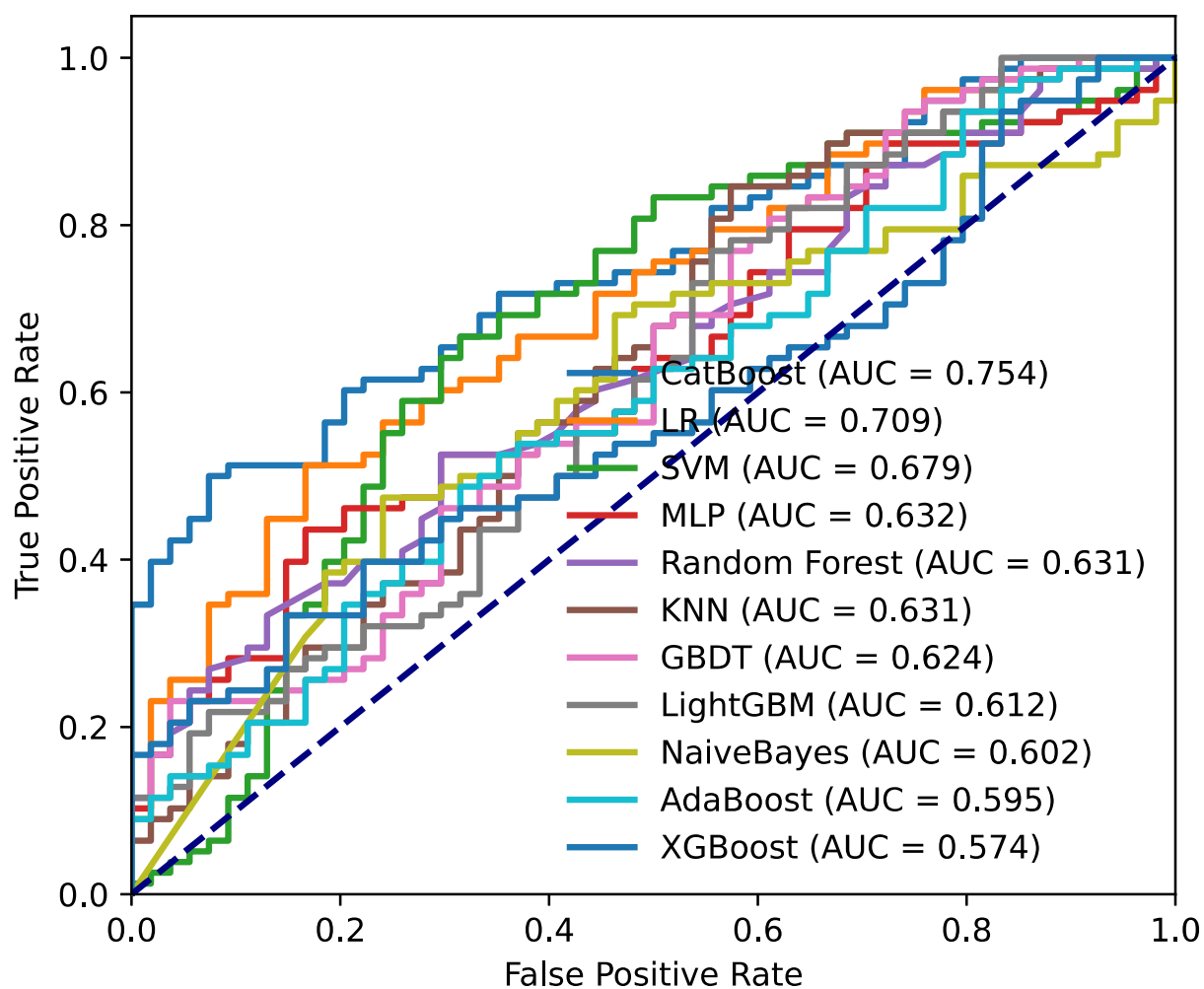

Supplement: Supplementary Figure 2 — Receiver operating characteristic curves for the machine learning model and logistic regression in the validation set. [file DataSheet_2.pdf]

Train ROC Curve

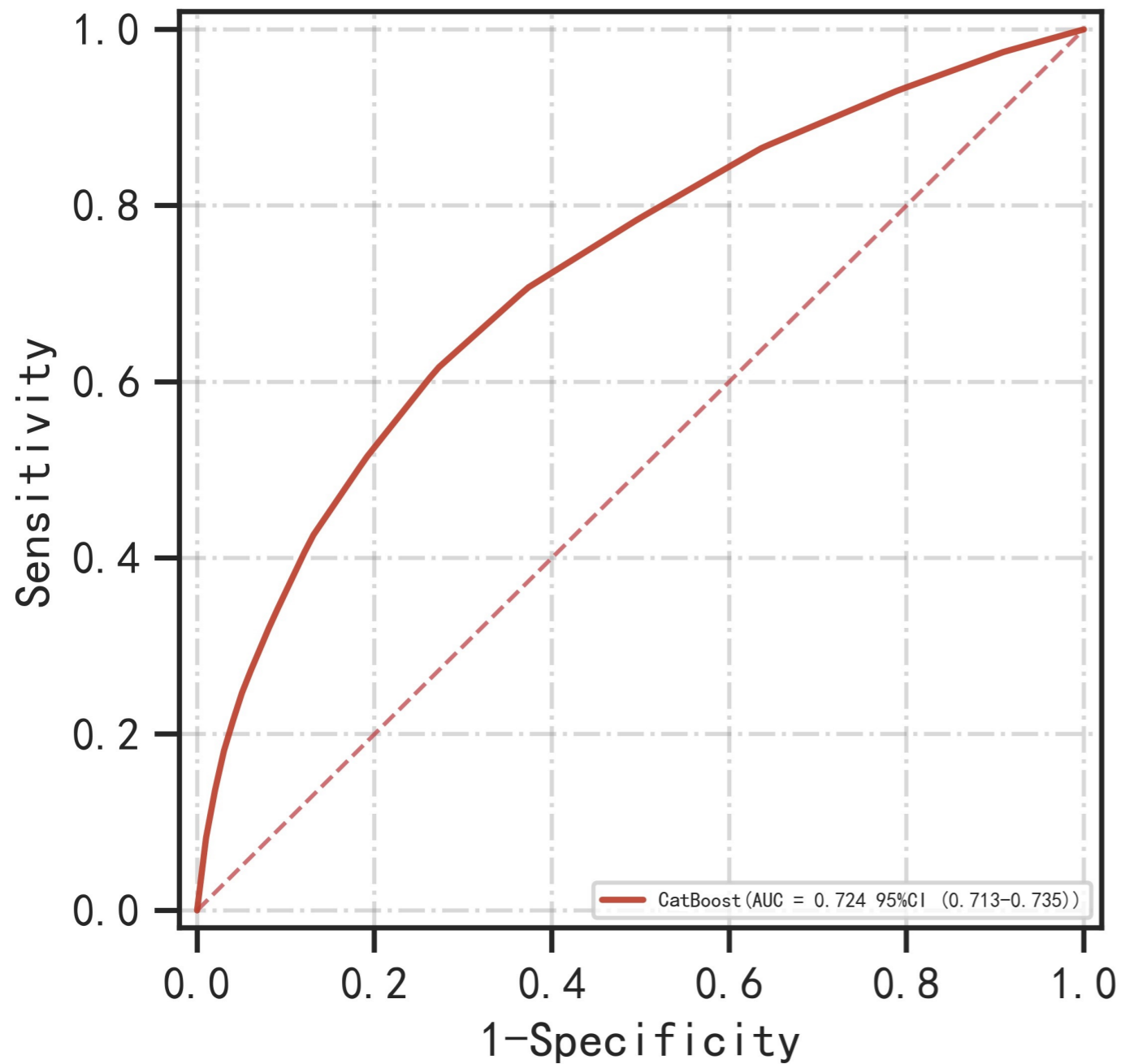

Validation ROC Curve

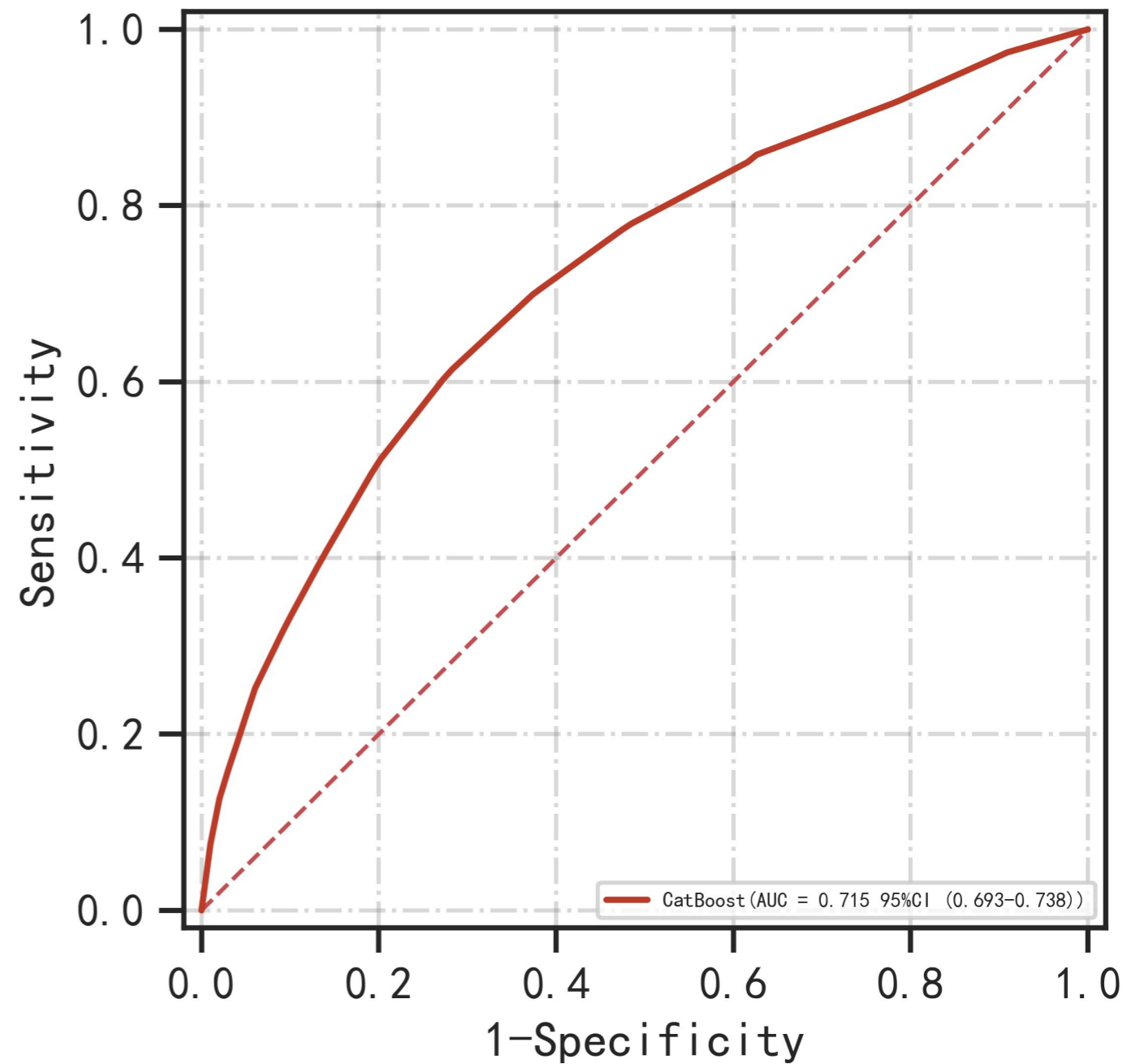

Supplement: Supplementary Figure 3 — Receiver operating characteristic curves for the machine learning model of SOFA score. (A) ROC of the training set. (B) ROC of the validation set. [file DataSheet_3.pdf]

### Train ROC Curve

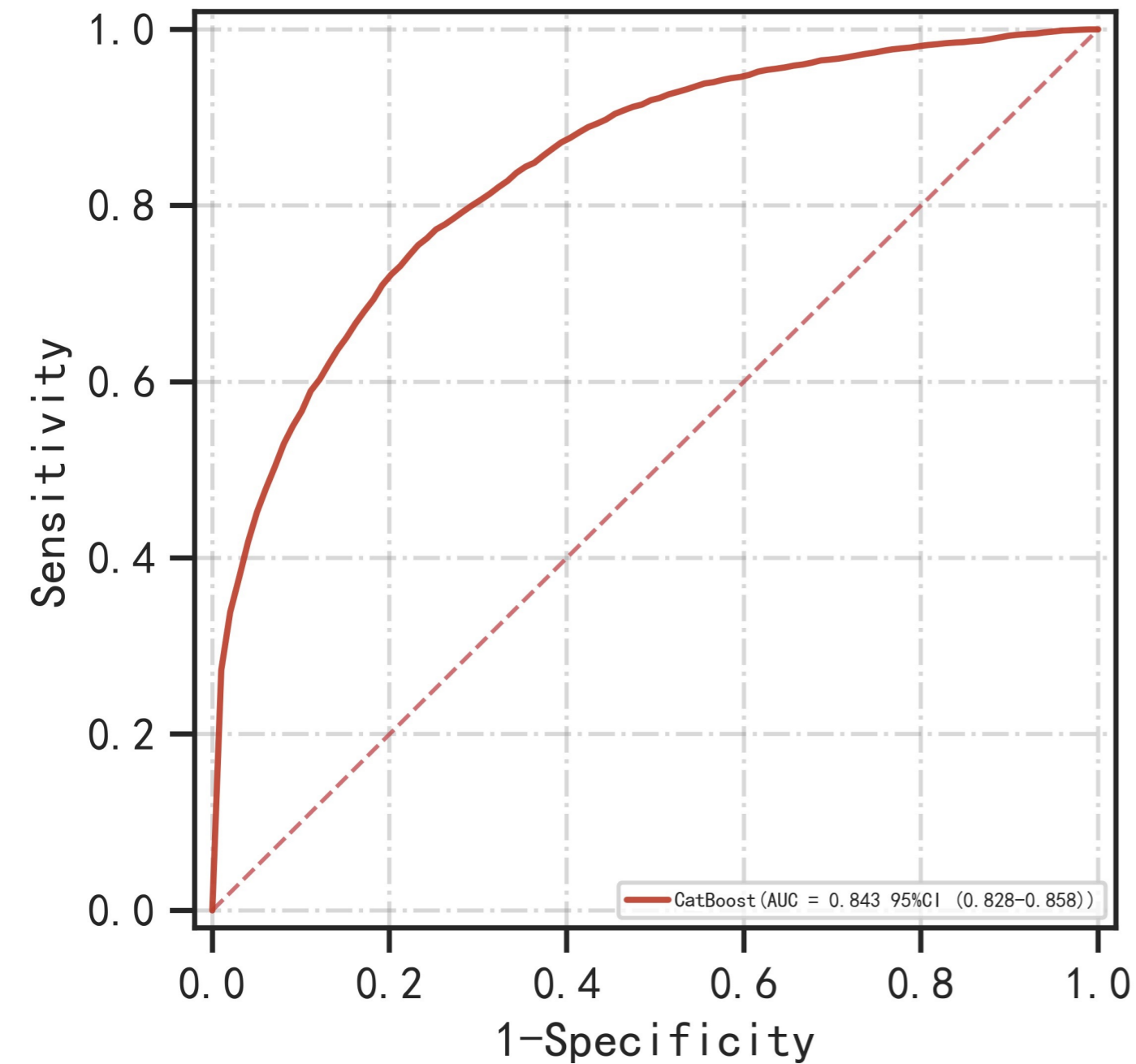

### Validation ROC Curve

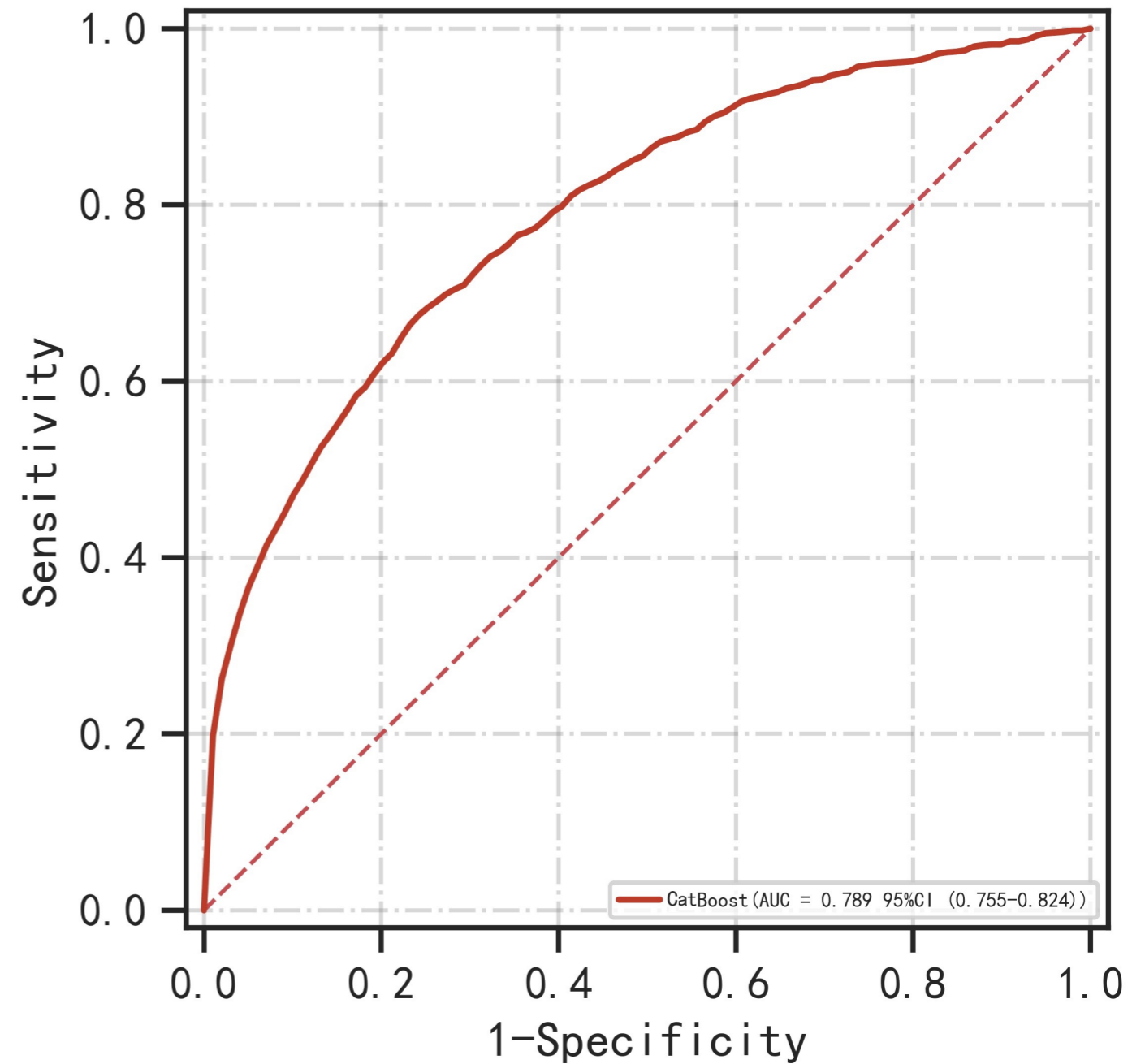

Supplement: Supplementary Figure 4 — Receiver operating characteristic curves for the machine learning model of liver disease subgroup. (A) ROC of the training set. (B) ROC of the validation set. [file DataSheet_4.pdf]
