# Supplementary material for: Machine learning for the prediction of all-cause mortality in patients with sepsis-associated acute kidney injury during hospitalization
Source: Front Immunol. 2023 Apr 3;14:1140755. doi: 10.3389/fimmu.2023.1140755 (PMC10106833; doi:10.3389/fimmu.2023.1140755)
Supplement: Supplementary Table 1 — Demographic and comorbidity information on the external validation cohort. [file DataSheet_5.pdf]

Varibale		All	Alive group	death group
n		132	54	78
Age,mean(SD)		56.7（16.1）	56.0（16.8）	57.5（15.7）
Gender, n (%)	F	44(33.3)	14（25.9）	30（38.5）
	M	88(66.7)	40（74.1）	48（61.5）
Liver disease, n (%)	1	29（22.0）	9（16.7）	20（25.6）
Cardiovascular events, n (%)	1	25（18.9）	5（9.3）	20（25.6）
Stroke,n (%)	1	12(9.1)	6(11.1)	6(7.7)
diabetes	1	19(14.4)	10(18.5)	9(11.5)
